# Phylogeographic and population insights of the Asian common toad (*Bufo gargarizans*) in Korea and China: population isolation and expansions as response to the ice ages

**DOI:** 10.7717/peerj.4044

**Published:** 2017-11-28

**Authors:** Amaël Borzée, Joana L. Santos, Santiago Sánchez-RamÍrez, Yoonhyuk Bae, Kyongman Heo, Yikweon Jang, Michael Joseph Jowers

**Affiliations:** 1 Laboratory of Behavioural Ecology and Evolution, School of Biological Sciences, Seoul National University, Seoul, South Korea; 2 CIBIO/InBIO (Centro de Investigação em Biodiversidade e Recursos Genéticos), Universidade do Porto, Campus Agrario De Vairão, Portugal; 3 Department of Ecology and Evolutionary Biology, University of Toronto, Toronto, ON, Canada; 4 Department of Natural History, Royal Ontario Museum, Toronto, ON, Canada; 5 Academy of Life Science and Biotechnology, Hallym University, Chuncheon, South Korea; 6 College of Natural Science, Sangmyung University, Seoul, South Korea; 7 Department of Life Sciences, Division of EcoScience, Ewha Womans University, Seoul, South Korea; 8 National Institute of Ecology, Geumgang-ro, Maseo-myeon, Seocheon-gun, South Chungcheong Province, South Korea

**Keywords:** Phylogeography, *Bufo gargarizans*, Korea, Asia, China, Common Asian toad, Ice ages

## Abstract

The effects of ice ages on speciation have been well documented for many European and North American taxa. In contrast, very few studies have addressed the consequences of such environmental and topographical changes in North East Asian species. More precisely, the Korean Peninsula offers a unique model to assess patterns and processes of speciation as it hosts the northern- and eastern-most distribution limit of some widespread Asian taxa. Despite this, studies addressing phylogeographic patterns and population genetics in the peninsula and surrounding countries are few and studies for most families are lacking. Here we inferred the phylogenetic relationships of the common toad (*Bufo gargarizans*) from South Korea and their North East Asian counterpart populations, based on mitochondrial data. Korean *B. gargarizans* GenBank BLASTs matched few individuals from nearby China, but the presence of a Korean clade suggests isolation on the Korean Peninsula, previous to the last glacial maximum, linked to sea level resurgence. Molecular clock calibrations within this group were used to date the divergence between clades and their relationship to paleo-climatic events in the area. Lack of genetic structure among South Korean populations and strong homogeneity between the Korean and some Chinese localities suggest weak isolation and recent expansion. Geographical projection of continuous coalescent maximum-clade-credibility trees shows an original Chinese expansion towards the Korean Peninsula through the Yellow Sea circa two million years ago with colonisation events dating circa 800 thousand years ago (K. y. a.). Following this colonisation, the data point to outgoing Korean Peninsula dispersal events throughout different periods, towards the North through land, and West through land bridge formations over the Yellow Sea during sea level falls. In accordance, demographic analyses revealed a population expansion in the Koran Peninsula circa 300 K. y. a., likely attributed to glacial cycle fluctuations.

## Introduction

Populations contract and expand throughout periods of glacial maxima and minima, respectively. Such harsh environmental fluctuations result in specific speciation events, and have been well documented across Europe (Hewitt, 2000; Veith, Kosuch & Vences, 2003; Avise, 2007) and North America (Knowles, 2001). Specifically, the last glacial maximum (LGM) in Europe resulted in speciation patterns following the isolation of populations on southern peninsulas, which were then used as refugia from colder and dryer conditions. Such an example is the *Hyla arborea* complex (Stöck et al., 2008, 2012). In contrast, evolutionary responses to climate fluctuations have been poorly studied in North East Asia, defined as the region north of the Yangtze River and limited around 100° West, due to the paleo-geographic coherence and the same monsoon climate regime of the region. However, studies suggests that rather than isolating populations throughout the LGM, they seem to have aided connectivity at different times by the drainage of the Yellow Sea resulting in land-bridge formations between the mainland, the current Korean Peninsula and the Japanese archipelago (Fairbanks, 1989; Park, Khim & Zhao, 1994; Kim & Kennett, 1998; Ijiri et al., 2005). The inter-glacial periods resulted in island discontinuity, due to sea level resurgence. Several hypothesis have been tested (see Kim et al., 2013), in relation to the potential for several refugia, the connectivity of the Korean Peninsula and the Asian mainland through land bridges, and the presence of ice sheets during the glacial oscillations. It seems certain that several refugia were present in North East Asia (Aizawa, Kim & Yoshimaru, 2012) during glacial or interglacial periods.

The *Bufo* genus (*sensu stricto*) is composed of two groups, the European–African–Western Asian group: ‘*Bufo bufo* group’ and the Eastern Asian ‘*Bufo gargarizans* group’ (Garcia-Porta et al., 2012). The *B. gargarizans* group ranges from Japan to south-western China and northern Vietnam, through south eastern Russia (Garcia-Porta et al., 2012), and includes the species *B. gargarizans*, *Bufo bankorensis* and *Bufo japonicus*. The species with the largest range, the Asian toad (*B. gargarizans*), ranges from Japan to China (Kuzmin et al., 2004) as a single species despite multiple contested lineages (Liu et al., 2000; Zhan & Fu, 2011), and east-west genetic dispersion patterns (Fu et al., 2005). The low genetic divergence throughout its range is likely a result of a lack of an isolated insular refugium (Zhan & Fu, 2011; Yan et al., 2013). Another factor to the genetic homogeneity of the species is the absence of mountain chains high enough to act as barriers within the species range. Instead, the Himalayan range, bordering the southern edge of the Tibetan plateau, sets a southern edge to the distribution of the species (Yan et al., 2013). Other studies however, focusing on North East Asian mainland *B. gargarizans* populations, show a contrasting picture with some species showing genetic isolation, such as isolated peninsular or insular populations. This is demonstrated through the dichotomy between *B. gargarizans* and *B. bankorensis*, with the first species on the mainland and the latter in Taiwan (Chen et al., 2013; Yu, Lin & Weng, 2014). Coastal North East Asia was potentially composed of several refugia during either glacial or interglacial periods, thus reducing gene flow connectivity, even for good dispersers such as raccoon dogs (Kim et al., 2013). The Korean Peninsula was not covered by glaciers, but it was colder and drier during glacial periods (Kong, 2000; Yi & Kim, 2010). It was also a refugium during the late Pleistocene, as exemplified for *Pelophylax nigromaculatus* (Zhang et al., 2008) and *Onychodactylus fischeri* (Yoshikawa et al., 2008). Moreover, the Korean Peninsula is divided by geographical landscape barriers, leading to the emergence of several clades within *Pelophylax chosenicus* (Min et al., 2008), *Hynobius* spp. (Baek et al., 2011; Min et al., 2016) and *Dryophytes japonicus* (Dufresnes et al., 2016), a consequence of allopatric divergence.

**Figure 1:**
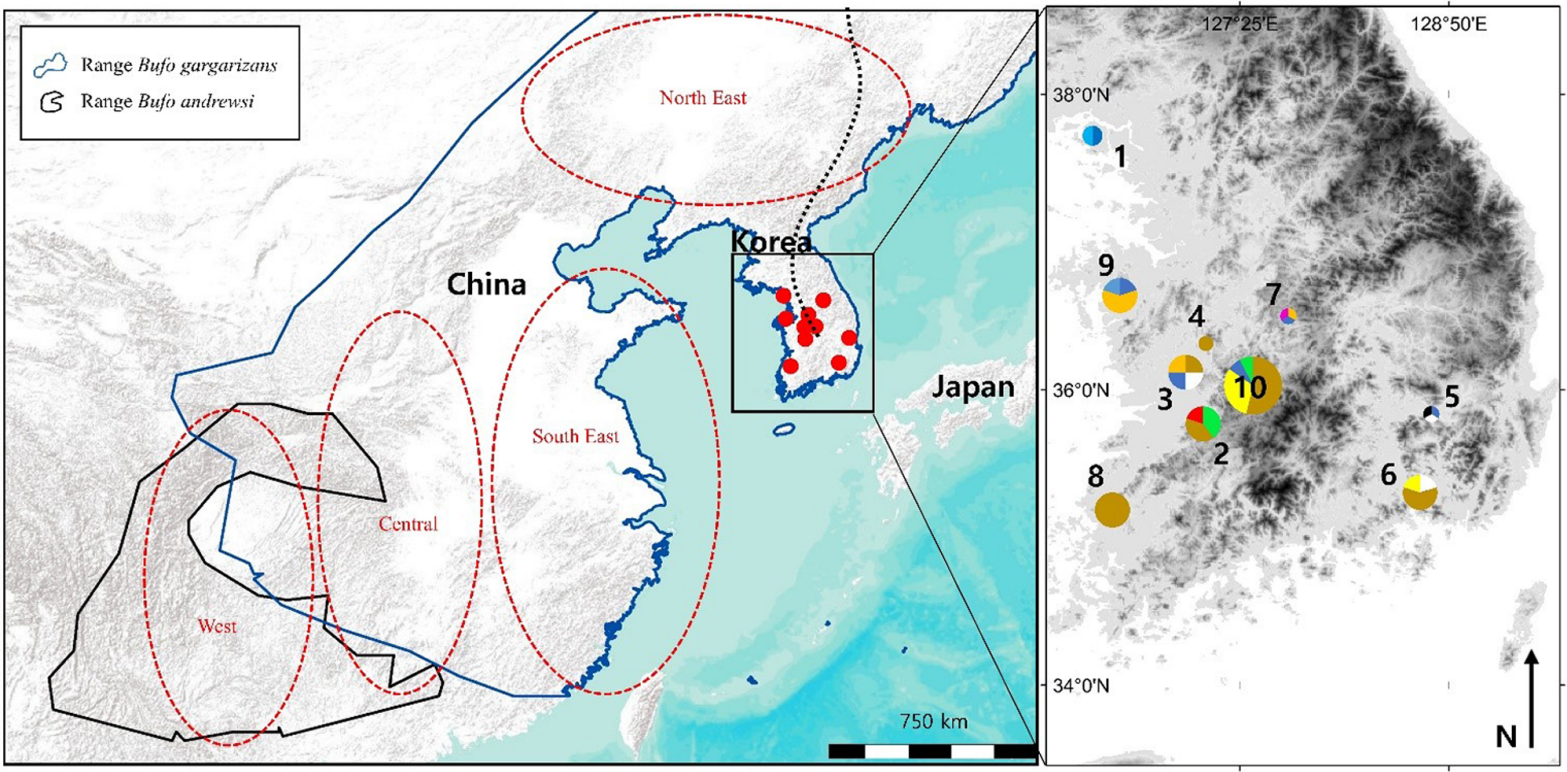
Map of the sampling sites where *Bufo gargarizans* roadkills were collected and regions of origin for the samples extracted from GeneBank. On the inset map, each colour matches with a different haplotype of the combined analysis, encoded similarly to the haplotype networks (Fig. 5C). The size of the pie charts is proportional to the sample size at the site (1 < *n* < 13). Site 1 is on Gangwha Island; and the black dashed line represents the Baekdu Mountains Range. This figure was generated with Google Earth Pro (Mountain View, CA, USA) and ArcMap 10.5 (Environmental Systems Resource Institute, Redlands, CA, USA). Service Layer Credits & Sources: Esri, USGS and GeoServices Map Esri Korea). Map data ©2015 Google.

Connectivity between the Korean Peninsula and the rest of the Eurasian continent has been regularly ensured by the filling of the Yellow Sea, even during glaciation periods when the northern latitude of the peninsula was not within amphibian reach, and thus through continued land connectivity (Haq, Hardenbol & Vail, 1987; Millien-Parra & Jaeger, 1999; see Fig. 1, Kim et al., 2013). However, genetic diversification between China, Korea and Japan occurred (Ota, 1998; Ota et al., 2002; Zhang et al., 2008; Gong et al., 2008; Qi et al., 2014; Matsui, 2014), exemplified by repeated patterns of mtDNA introgression and range shifts in water frogs (*Pelophylax* spp.; Komaki et al., 2015).

*Bufo gargarizans* populations have shown a slow decline in Korea throughout the last decades, mostly due to habitat partitioning (Kuzmin et al., 2004). The genetic structure of populations in North East Asia is fairly well known for Chinese (Liu et al., 2000; Fu et al., 2005; Zhan & Fu, 2011), Taiwanese (Chen et al., 2013; Yu, Lin & Weng, 2014) and Japanese (Hase, Shimada & Nikoh, 2012) populations, but very little is known so far for Korean populations (Fu et al., 2005). Herewith, we hypothesise the absence of a total genetic segregation within *B. gargarizans* populations from South Korea due to the potential for recolonisation and population connectivity during interglacials.

## Materials and Methods

### Field work

*Bufo gargarizans* is usually found between 20 and 800 m a.s.l., in coniferous, mixed and deciduous forests, as well as grasslands, in humid but not saturated habitat (Kuzmin et al., 2004). Due to permit restriction from the Ministry of Environment (permit numbers: Yesan-2016-10; Boeun-2016-01; Gangwha-2016-01; Jeonju-2016-01; Hampyeong-2016-37; Daegu-2016-01; Geumsan-2016-02; Nonsan-2016-01; Changwon-2016-02) we limited our sampling to 47 individuals from 10 sites (Fig. 1), with all samples originating from road kills. For samples to be assigned to a same localities, they had to be collected within 10 km of each other, representative of an average maximum dispersion distance for amphibians (see review by Smith & Green, 2005). To define the GPS coordinates of the localities for pooled samples (Table 1), we calculated the centre of gravity created by the polygon of sites considered, weighted by the number of samples.

**Table 1:** Sampling localities for all 47 individuals included in this study.

Site ID on Fig. 1	Country	Latitude (N)	Longitude (W)	Sample size	Sample ID	GenBank Accession Number		Origin
		(or localities)				CR	ND2	
1	Korea	37.721666°	126.401131°	2	*B. gargarizans* 1	KY295993	KY295992	This study
1	Korea	37.721666°	126.401131°	2	*B. gargarizans* 2	KY295994	KY295991	This study
2	Korea	35.779648°	127.143229°	5	*B. gargarizans* 3	KY295995	KY295990	This study
2	Korea	35.779648°	127.143229°	5	*B. gargarizans* 4	KY295996	KY295989	This study
2	Korea	35.779648°	127.143229°	5	*B. gargarizans* 5	KY295997	KY295988	This study
2	Korea	35.779648°	127.143229°	5	*B. gargarizans* 6	KY295998	KY295987	This study
2	Korea	35.779648°	127.143229°	5	*B. gargarizans* 7	KY295999	KY295986	This study
3	Korea	36.137497°	127.381469°	5	*B. gargarizans* 8	KY296000	KY295985	This study
3	Korea	36.137497°	127.381469°	5	*B. gargarizans* 9	KY296001	KY295984	This study
3	Korea	36.137497°	127.381469°	5	*B. gargarizans* 10	KY296002	KY295983	This study
3	Korea	36.137497°	127.381469°	5	*B. gargarizans* 11	KY296003	KY295982	This study
3	Korea	36.137497°	127.381469°	5	*B. gargarizans* 12	KY296004	KY295981	This study
4	Korea	36.310532°	127.185360°	1	*B. gargarizans* 13	KY296005	KY295980	This study
5	Korea	35.825662°	128.704850°	3	*B. gargarizans* 14	KY296006	KY295979	This study
5	Korea	35.825662°	128.704850°	3	*B. gargarizans* 15	KY296007	KY295978	This study
5	Korea	35.825662°	128.704850°	3	*B. gargarizans* 16	KY296008	KY295977	This study
6	Korea	35.291573°	128.672657°	5	*B. gargarizans* 17	KY296009	KY295971	This study
6	Korea	35.291573°	128.672657°	5	*B. gargarizans* 18	KY296010	KY295976	This study
6	Korea	35.291573°	128.672657°	5	*B. gargarizans* 19	KY296011	KY295975	This study
6	Korea	35.291573°	128.672657°	5	*B. gargarizans* 20	KY296012	KY295974	This study
6	Korea	35.291573°	128.672657°	5	*B. gargarizans* 21	KY296013	KY295973	This study
7	Korea	36.485968°	127.737251°	3	*B. gargarizans* 22	KY296014	KY295972	This study
7	Korea	36.485968°	127.737251°	3	*B. gargarizans* 23	KY296015	KY295970	This study
7	Korea	36.485968°	127.737251°	3	*B. gargarizans* 24	KY296017	KY295969	This study
8	Korea	35.181633°	126.541167°	5	*B. gargarizans* 25	KY296018	KY295968	This study
8	Korea	35.181633°	126.541167°	5	*B. gargarizans* 26	KY296019	KY295967	This study
8	Korea	35.181633°	126.541167°	5	*B. gargarizans* 27	KY296020	KY295966	This study
8	Korea	35.181633°	126.541167°	5	*B. gargarizans* 28	KY296021	KY295965	This study
8	Korea	35.181633°	126.541167°	5	*B. gargarizans* 29	KY296016	KY295964	This study
9	Korea	36.666582°	126.642740°	5	*B. gargarizans* 30	KY296022	KY295963	This study
9	Korea	36.666582°	126.642740°	5	*B. gargarizans* 31	KY296023	KY295962	This study
9	Korea	36.666582°	126.642740°	5	*B. gargarizans* 32	KY296024	KY295961	This study
9	Korea	36.666582°	126.642740°	5	*B. gargarizans* 33	KY296025	KY295960	This study
9	Korea	36.666582°	126.642740°	5	*B. gargarizans* 34	KY296026	KY295959	This study
10	Korea	36.082754°	127.494845°	13	*B. gargarizans* 35	KY296027	KY295958	This study
10	Korea	36.082754°	127.494845°	13	*B. gargarizans* 36	KY296028	KY295957	This study
10	Korea	36.082754°	127.494845°	13	*B. gargarizans* 37	KY296029	KY295956	This study
10	Korea	36.082754°	127.494845°	13	*B. gargarizans* 38	KY296030	KY295955	This study
10	Korea	36.082754°	127.494845°	13	*B. gargarizans* 39	KY296031	KY295954	This study
10	Korea	36.082754°	127.494845°	13	*B. gargarizans* 40	KY296032	KY295953	This study
10	Korea	36.082754°	127.494845°	13	*B. gargarizans* 41	KY296033	KY295952	This study
10	Korea	36.082754°	127.494845°	13	*B. gargarizans* 42	KY296034	KY295951	This study
10	Korea	36.082754°	127.494845°	13	*B. gargarizans* 43	KY296035	KY295950	This study
10	Korea	36.082754°	127.494845°	13	*B. gargarizans* 44	KY296036	KY295949	This study
10	Korea	36.082754°	127.494845°	13	*B. gargarizans* 45	KY296037	KY295948	This study
10	Korea	36.082754°	127.494845°	13	*B. gargarizans* 46	KY296038	KY295947	This study
10	Korea	36.082754°	127.494845°	13	*B. gargarizans* 47	KY296039	KY295946	This study
CIB-XM	China	Antu		1	CIBX-M014	AY924344	AY936870	Fu et al. (2005)
CIB-XM	China	Antu		1	CIBX-M076	AY924345	AY936867	Fu et al. (2005)
HB	China	Hubei Shishou		5	HB2^1^	DQ288717	–	Hu et al. (2007)
HB	China	Hubei Shishou		5	HB4^1^	DQ288717	–	Hu et al. (2007)
HB	China	Hubei Shishou		5	HB5^1^	DQ288717	–	Hu et al. (2007)
HB	China	Hubei Shishou		5	HB6^1^	DQ288717	–	Hu et al. (2007)
HB	China	Hubei Shishou		5	HB7^1^	DQ288717	–	Hu et al. (2007)
GS	China	Gansu Lanzhou		5	GS41^3^	DQ288719	–	Hu et al. (2007)
GS	China	Gansu Lanzhou		5	GS46^3^	DQ288719	–	Hu et al. (2007)
GS	China	Gansu Lanzhou		5	GS47^3^	DQ288719	–	Hu et al. (2007)
GS	China	Gansu Lanzhou		5	GS48^3^	DQ288719	–	Hu et al. (2007)
GS	China	Gansu Lanzhou		5	GS410^1^	DQ288717	–	Hu et al. (2007)
HLJ	China	Heilongjiang Harbin		3	HLJ1^1^	DQ288717	–	Hu et al. (2007)
HLJ	China	Heilongjiang Harbin		3	HLJ2^2^	DQ288718	–	Hu et al. (2007)
HLJ	China	Heilongjiang Harbin		3	HLJ3^2^	DQ288718	–	Hu et al. (2007)

**Notes:**

The GPS coordinates provided are the centre of gravity calculated from the different sampling sites. All sampling sites within 10 km of each other were considered a single locality. The samples downloaded from GenBank grouped within the Korean clade are listed in the table.

Superscripts (^1,2,3^) by sample ID codes denote same CR haplotypes.

### Molecular work

**Figure 2:**
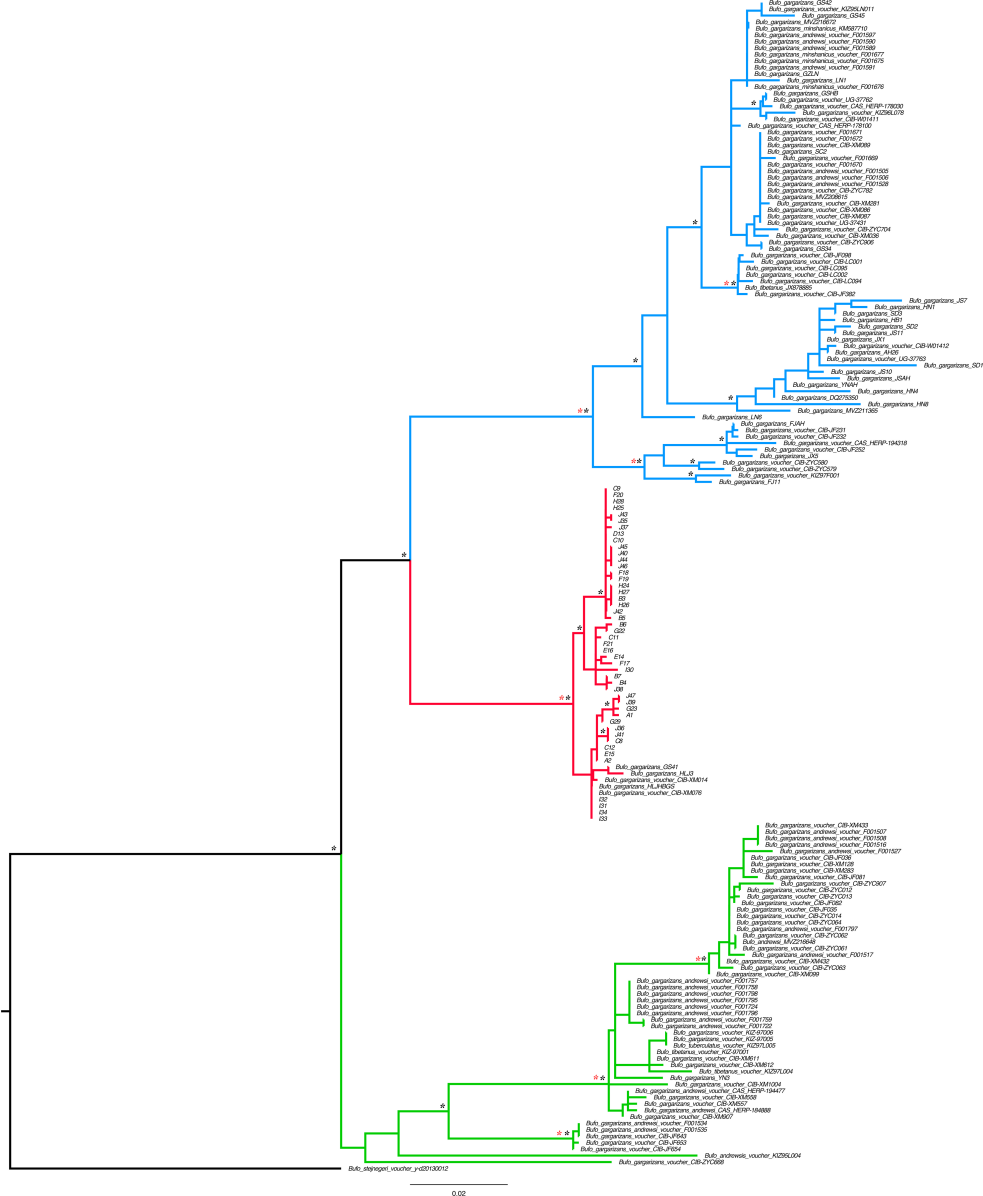
Best Maximum Likelihood tree (*n* = 181) recovered from the RAxML analyses and BI for the ND2 and CR fragments, displaying the genetic structure of *Bufo gargarizans* from the Korean Peninsula and mainland Chinese localities. Posterior probabilities > 0.95 from the Bayesian Inference are indicated on nodes as * in black (BI) in red (ML). Green clade; Western China, red clade; Korea (+ 5 Chinese haplotypes), blue clade; all Chinese localities.

**Figure 3:**
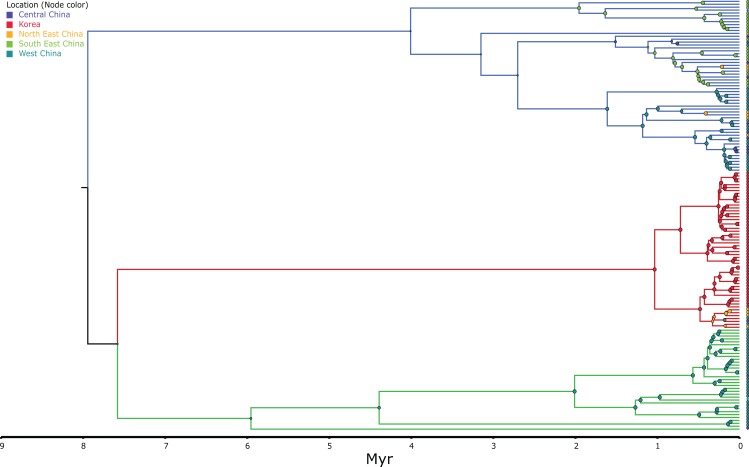
MCC discrete coalescent tree (*n* = 148) of *Bufo gargarizans*. Node circle are colour coded by localities and sizes correspond to probability of the locality origin. Green clade; Western China, red clade; Korea (+ 5 Chinese haplotypes), blue clade; all Chinese localities.

**Figure 4:**
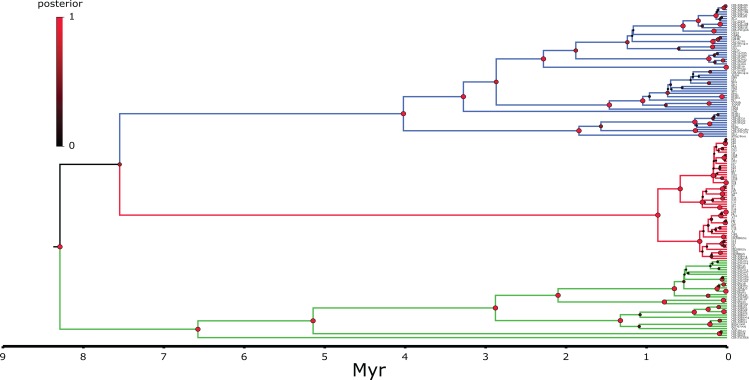
MCC continuous coalescent tree (*n* = 148) of *Bufo gargarizans*. High posterior probability nodes are represented in red circles. Green clade; Western China, red clade; Korea (+ 5 Chinese haplotypes), blue clade; all Chinese localities.

DNA was extracted through a Qiagen DNeasy blood and tissue kit (Qiagen, Hilden, Germany) following the instructions of the manufacturer. The targeted genes were the mitochondrial ND2 (this fragment comprised 74 bp ND1, complete tRNA-Ile, tRNA-Gln, tRNA-Met, 510 bp ND2; referred throughout as ND2 fragment for simplicity) and CR fragments. The primers used were: ND2: L-int 5′-CGA GCA TCC TAC CCA CGA TTT CG-3′ (Fu et al., 2005) and H4980 5′-ATT TTT CGT AGT TGG GTT TGR TT-3′ (Macey et al., 1998) and CR BGarF 5′-TTGGACGATAGCAAGGAACACTC-3′ and CR BGarR 5′-CCTGACTTCTCTGAGGCCGCTTT-3′ (this study). Templates were sequenced on both strands and the complementary reads were used to resolve rare, ambiguous base-calls in Sequencher v.4.9. In order to assess the positioning of the Korean individuals within the *B. gargarizans* species complex, several alignments and analyses were prepared depending on localities and GPS coordinates in Genbank records. A larger alignment for the RAxML and MrBayes analyses consisted of all the *B. gargarizans* specimens available in Genbank and other closely related taxa that fell within the *B. gargarizans* species complex, not associated to additional data such as exact geographical localities (*n* = 181, length = 1,222 base pair = bp, Fig. 2). A slightly smaller alignment that included individuals assigned to *B. gargarizans* senso strito that either had GPS or accurate Chinese localities (*n* = 148, CR = 427 bp, ND2 = 795, bp = 1,222, Figs. 3 and 4) was used to perform statistical coalescent and phylogeographic analyses (see below). Lastly, we built a smaller alignment that only included all the Korean *B. gargarizans* (*n* = 47) form Korea and all closely related matches from nearby China (*n* = 15; Fig. 6; Figs. S1 and S2). The length of this alignment was 422 and 795 bp for the CR and ND2 fragments, respectively. The combined data set resulted in 1,217 bp in length.

**Figure 5:**
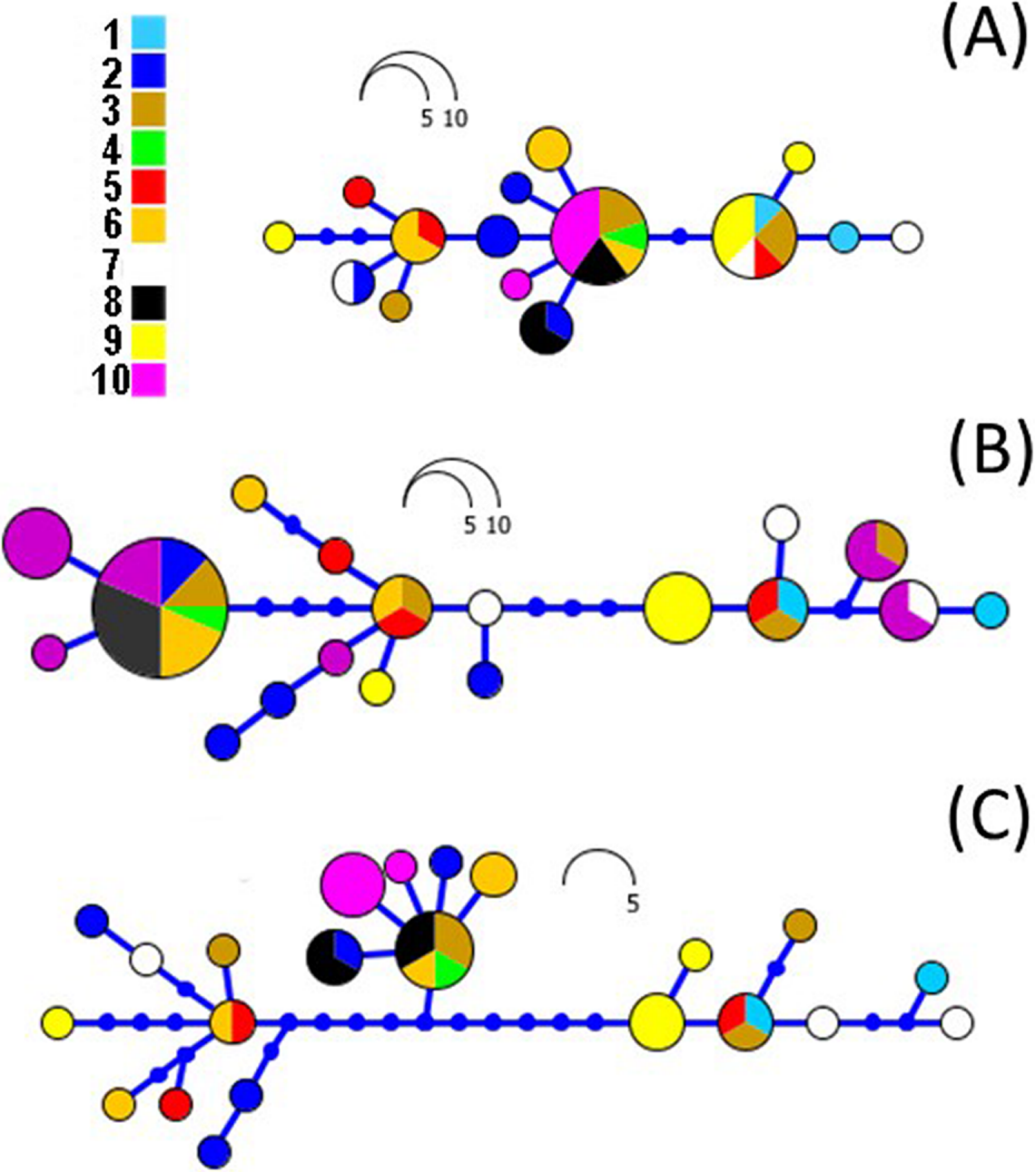
RAxML networks based on ND2 (A), CR (B) and combined datasets (C). Networks were built on Haploviewer under the best tree topology as inferred in RAxML. Numbers 1–10 relate to the coding of populations from Table 1 and Fig. 1.

Sequences were aligned in Seaview v.4.2.11 (Gouy, Guindon & Gascuel, 2010) under ClustalW2 (Larkin et al., 2007) with default settings. Genetic *p*-distances (pairwise deletion) and standard error (% ± SE) were calculated using Mega 7 (Kumar, Stecher & Tamura, 2016). The most appropriate substitution model for the Bayesian inference (BI) analysis was determined by the Bayesian information criterion (BIC) in PartitionFinder v.2 (Lanfear et al., 2012). MrBayes v.3.2.6 (Ronquist & Huelsenbeck, 2003) was used with default priors and Markov chain settings, and with a random starting tree. Each run consisted of four chains of 20,000,000 generations (small alignment, *n* = 62, Figs. S1 and S2), 100,000,000 (large alignment, *n* = 181, Fig. 2) sampled each 2,000 generations (small alignment) and 10,000 (large alignment), respectively. For the BI analyses, a plateau was reached after few generations with 25% of the trees resulting from the analyses discarded as burn in. Phylogenetic relationships among haplotypes for each locus were estimated using a maximum likelihood (ML) approach, as implemented in the software RAxML v7.0.4 (Silvestro & Michalak, 2012; Stamatakis, 2014), using the default settings. Node support was inferred by the bipartition method in RAxML with 10 random addition replicates. All analyses were performed through the CIPRES platform (Miller, Pfeiffer & Schwartz, 2010). Candidate outgroup species for the trees were *B. japonicus, Bufo stejnegeri* and *Bufo tibetanus*. However, divergence of *B. japonicus* and *B. tibetanus* were low and closely grouped within the *B. gargarizans* clades. Thus, we employed *B. stejnegeri* as the most suitable outgroup for the RaxML and MrBayes analyses of the larger data set (*n* = 181) and used *B. tibetanus* in the smaller data set (*n* = 63). Networks were built on Haploviewer (Salzburger, Ewing & Von Haeseler, 2011) under the best tree topology as inferred in RAxML for sequenced Korean individuals (Fig. 5). The molecular clock test was done in MEGA 7 (Kumar, Stecher & Tamura, 2016), using the entire small dataset (CR+ND2 concatenated 63 samples) and applying the GTR+G model (the same used in RAxML analyses). The Partition Homogeneity Test was performed in PAUP 4 (Swofford 2003; Tamura et al., 2013), applying the partition of each gene, CR+ND2.

**Figure 6:**
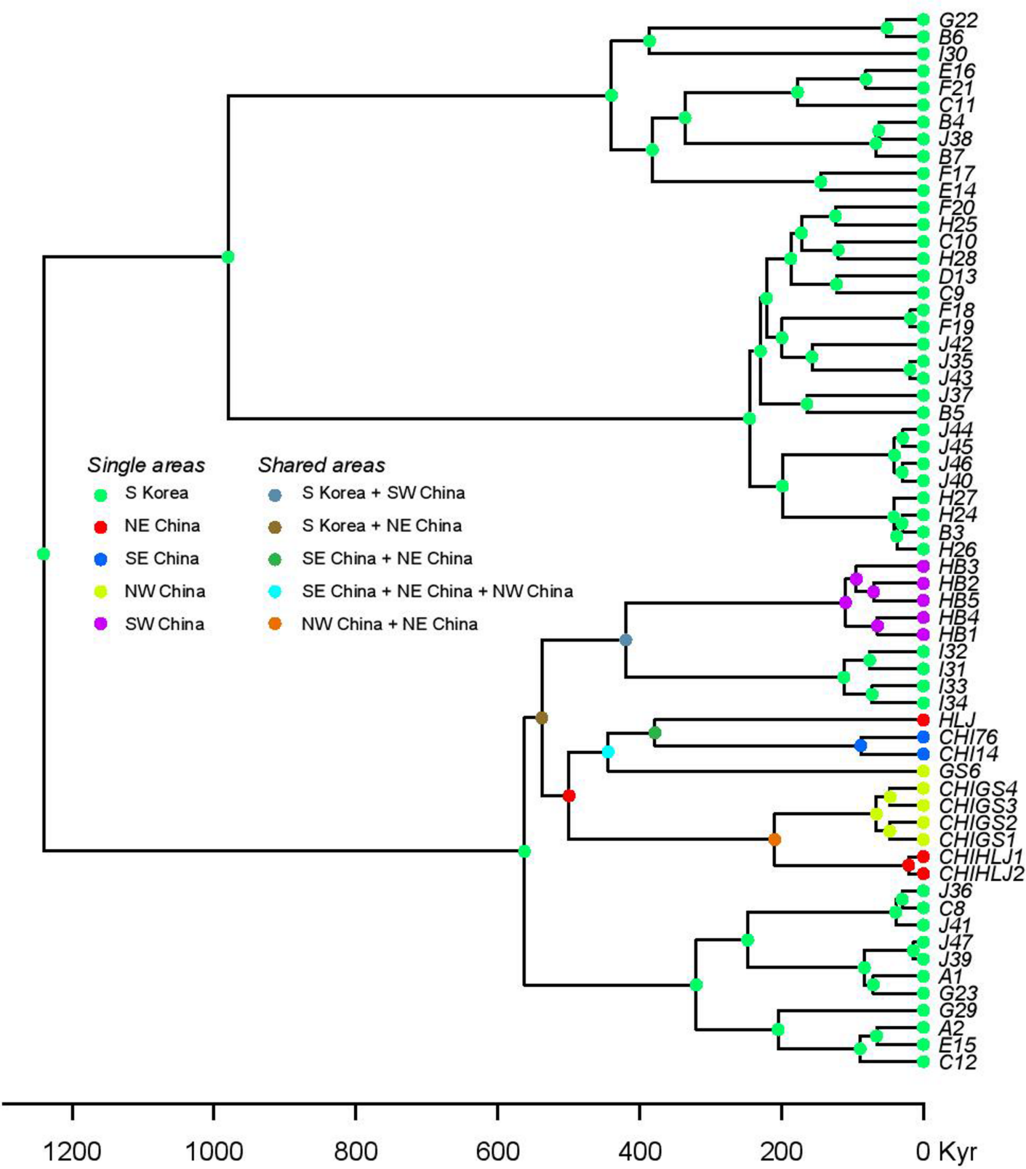
Statistical dispersal-vicariance analysis (S-DIVA) tree for all Korean and closely related Chinese *B. gargarizans*. Chinese localities refer to those in Fig. 7; Fig. S1.

In order to time-calibrate the population tree, we fixed the mutation rate in the CR to 7.3 × 10^−9^ substitutions/site/year following (Stöck et al., 2006) and the ND2 rate estimated from the prior. This CR rate lies as a mean from previous estimates from European *Bufo* genus species and *B. gargarizans*, between 0.067 and 0.087 per lineage per million years (Stöck et al., 2006) and are established based on the last connection between North Africa and Sicily about 5.3 million years ago (M. y. a.). For population size, we used a prior normal distribution with mean = 7.3 × 10^−9^ and standard deviation = 0.5. In addition to the population tree, we co-estimated the dispersal history using a discrete phylogeographic (ancestral state reconstruction) model (Lemey et al., 2009) implemented in BEAST v1.8.2 (Drummond et al. 2012; http://beast.bio.ed.ac.uk). Given that our geographic sampling of populations is uneven and our state-space is low, we chose a symmetric continuous-time rate matrix. As priors for the rates, we selected the approximate reference (CTMC) prior (Ferreira & Suchard, 2008). The discrete BI analyses (alignment *n* = 62) was run for 10 million generations and sampling every 1,000 generations. In addition, the statistical dispersal-vicariance analysis (S-DIVA) was run using RASP v3.0 (Yu et al., 2015), allowing all possible area overlaps and keeping a maximum of three areas per reconstructed node. Results were saved in text and PNG format and then edited by plotting the maximum-clade-credibility (MCC; see below) tree in *R* using APE (Paradis, Claude & Strimmer, 2004; Fig. 6).

**Figure 7:**
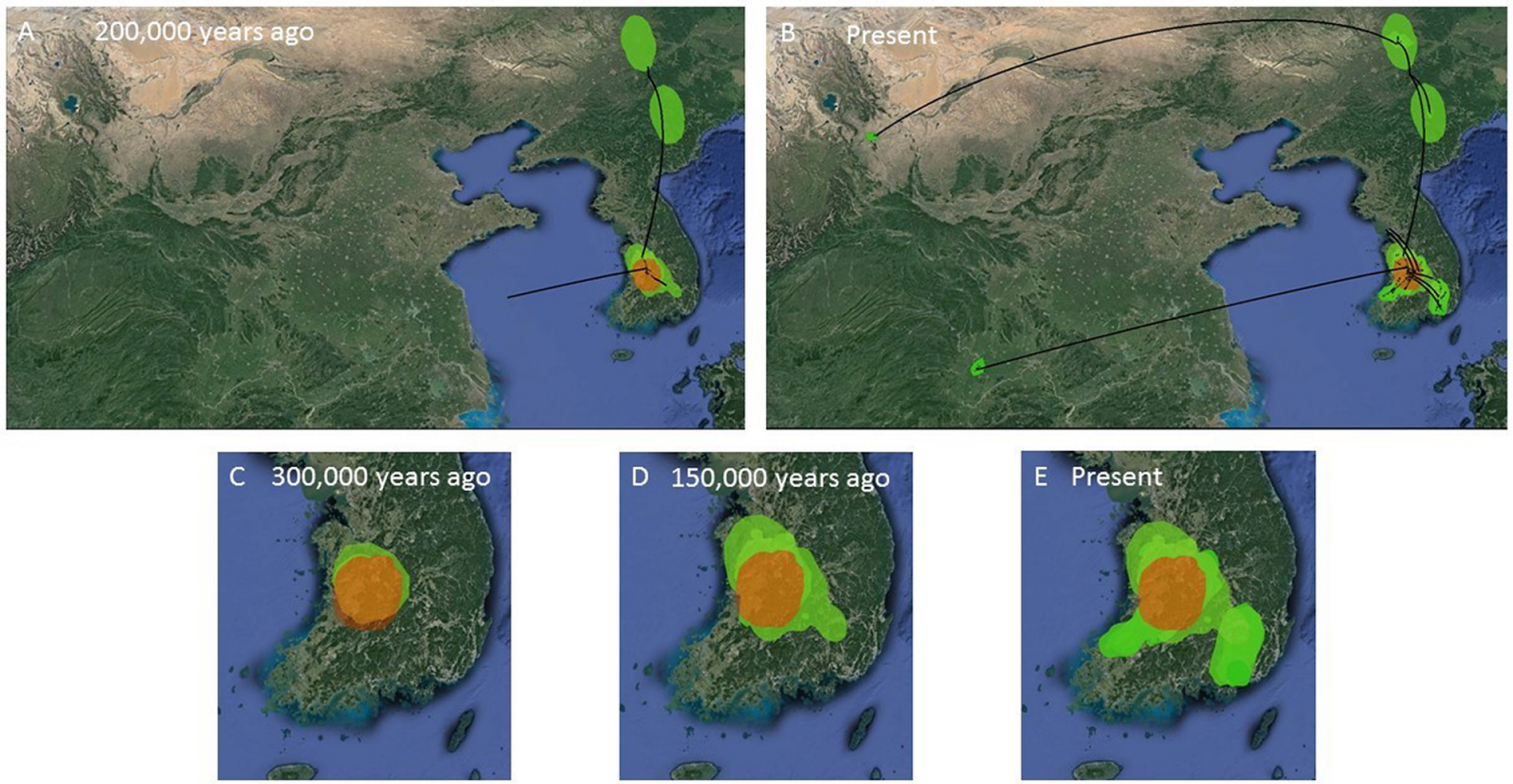
MCC Bayesian phylogeographic projections for *Bufo gargarizans* at different times scales. (A, B) Mainland expansions 200 K. y. a. and present, (C–E) Korean Peninsula expansions 300 K, 150 K. y. a., and present. The MCC gene tree is represented with black lines. Old diffusions shown in orange and more recent ones are shown in light green. Map data ©2015 Google.

**Figure 8:**
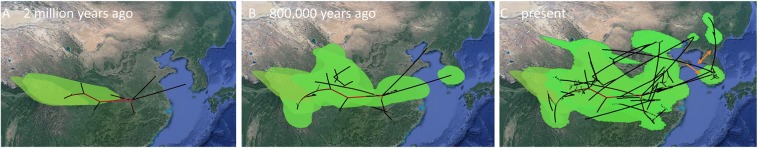
MCC Bayesian phylogeographic projections for *Bufo gargarizans* at different times scales for the complete data set of 148 individuals. (A) China mainland expansions 2 M. y. a, (B): China–Korea expansions 2 M. y. a.–800 K. y. a., (C) present expansions. The MCC gene tree is represented with black lines and old diffusions braches are shown in red. The orange arrows depict the three MCC branches of Korean to China expansions. Map data ©2015 Google.

Secondly, we estimated the diffusion of the *B. gargarizans* CR and ND2 sequence data through time using the continuous Bayesian phylogeographic approach (Lemey et al., 2010). This approach estimates population range changes through time and ancestral population locations or origins. We used the Gamma RRW model (alignment *n* = 62) and Cauchy RRW model (alignment *n* = 148) with all individuals assigned to GPS coordinates. A random ‘jitter’ was added to each GPS coordinate with a window size of 0.5. We applied a fixed clock rate to the CR partition (see above) and estimated from the prior for the ND2. We used marginal likelihood estimations (MLE) and Bayes factors (BF) to select for the continuous trait model. MLE were calculated through path sampling (PS) and stepping stone (SS) analyses in BEAST (Baele et al., 2012). We tested for Brownian, Gauchy, Gamma and Lognormal RRW models under the strict and relaxed lognormal models running 300 million generations, with sampling every 30,000 generations. The larger data set (*n* = 148) revealed chain convergence problems (e.g. Gamma RRW) with MLE analyses and therefore models were tested only on the strict clock. The posterior distributions of parameter estimates were visually inspected in Tracer v1.6 (Rambaut & Drummond, 2007). For all the models tested, MLE analyses were run for 50 path steps and 100,000 generations with each step. The BF were calculated as two times the difference in MLE between different models, and the significance was determined if the BF value was >10 (Kass & Raftery, 1995). Spatial continuous space MCC trees (*n* = 62 and *n* = 148) were computed in SPREAD (Bielejec et al., 2011) and viewed in Google Earth (http://earth.google.com; Figs. 7 and 8).

**Figure 9:**
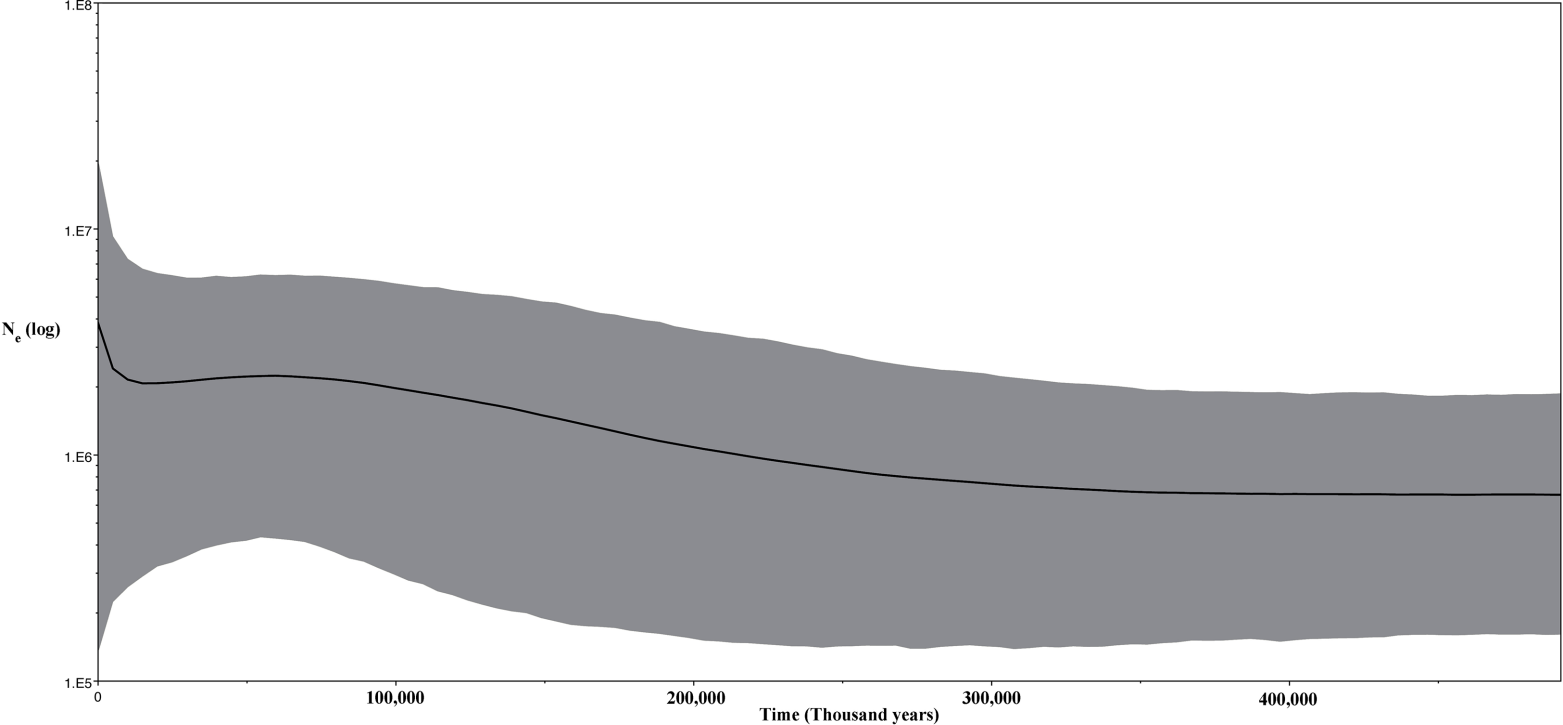
Bayesian skyline plot (BSP) for the ND2 and CR sequence data for Korean *Bufo gargarizans*.

Thirdly, we inferred changes in effective population size of the Korean individuals through time using a Bayesian skyline plot (BSP) model (Drummond et al., 2005) with strict clock for the CR data and same prior and a Lognormal relaxed clock for the ND2 fragment (Fig. 9). For both analyses, we ran two independent MCMC chains, each with 20 million states and sampling every 2,000 generations.

Independent runs were evaluated for convergence and mixing by observing and comparing traces of each statistic and parameter in Tracer v1.6 (http://beast.bio.ed.ac.uk/tracer). We considered effective sampling size (ESS) values >200 to be good indicators of parameter mixing. The first 10 states of the discrete, BSP and continuous Bayesian phylogenies were discarded as burnin. Samples were merged using LogCombiner v1.8.2. The resulting trees were summarised using TreeAnnotator v1.8.2, where a MCC tree with mean values was generated under heights = ca (Heled & Bouckaert, 2013). In the case of BSP, log and tree files were uploaded into Tracer, in which the plot was generated.

In addition, to examine whether the species showed any sign of historical population expansion, we estimated Tajima’s *D* (Tajima, 1989), Fu’s Fs (Fu, 1997) and a mismatch distribution analysis (Rogers & Harpending, 1992). Negative values of Tajima’s *D* can be interpreted as evidence of population expansions (Fu, 1997), and negative values of Fs indicate an excess of recent mutations and reject population stasis. In addition, we performed a Ramos-Onsins and Rozas (*R*^2^) analyses (Ramos-Onsins & Rozas, 2000). Diagrams of frequencies of pairwise genetic differences were drawn using DnaSP v.5.0 (Rozas et al., 2003). A thousand bootstrap replicates were used to generate an expected distribution using a model of sudden demographic expansion (Excoffier, Laval & Schneider, 2005). This mismatch distribution is usually multimodal in samples drawn from populations at demographic equilibrium, but is usually unimodal in populations following recent population demographic and range expansion (Slatkin & Hudson, 1991; Rogers & Harpending, 1992; Ray, Currat & Excoffier, 2003). Based on the lack of population differentiation between Korean (*n* = 47) and Chinese (*n* = 15) sequences were all considered as one population for demographic population analyses.

## Results

Preliminary phylogenetic data on all *B. gargarizans* from GenBank revealed intricate phylogenetic relationships mostly through paraphyly within the species complex. *Bufo tibetanus*, *B. bankorensis, B. japonicus, B. stejnegeri, B. andrewski* were closely associated to the Korean specimens generated from this study and to other Asian *B. lineages gargarizans*. Although out of the scope of this work, it is evident that this group is in need of taxonomical assessment. All trees recovered the Korean *B. gargarizans* as monophyletic with five Chinese haplotypes (*n* = 15) nested with in the Korean *B. gargarizans* clade (Figs. 2–4, 6; Figs. S1–S3) and were therefore included for the final phylogenetic analyses. GenBank BLAST searches of CR and ND2 sequences matched the same haplotype between Korean populations and three Chinese populations (Table 1). The ND2 and CR data set recovered 11 and 15 haplotypes, respectively. The molecular clock test (*p* = 0.999) confirmed that the null hypothesis could not be rejected. The Partition Homogeneity Tests (*p* = 0.90) indicated no significant heterogeneity between partitions and therefore analyses were performed with the concatenated alignment.

Uncorrected p-distances of haplotypes within the two major clades recovered by the phylogenetic analyses (Fig. 6; Figs. S1–S3) were low, CR (Korea + China clade; 0.51 ± 0.05%, Korean clade; 0.96 ± 0.07%), ND1 (Korea + China clade; 0.1 ± 0.02%, Korean clade; 0.33 ± 0.02%) and were not much different to the divergence between clades based on the ND2 fragment (ND1; 0.39 ± 0.01%) but lower than based on the CR clade divergence (CR; 1.7 ± 0.05%). The uncorrected p-distances between the Chinese and Korean individuals were low and again highlight the lack of genetic isolation between localities.

The unimodal mismatch distribution (Fig. S4) of the Korean and Chinese *B. gargarizans* suggests a population expansion (Fig. 9). Although the CR data set did not show statistical significance for demographic expansion, the overall negative Fu’s *F* and Tajima *D* for both markers (ND2, Fu’s *F* = −4.67, *p* = 0.006, Tajima’s *D* = −0.55 NS; CR, Fu’s *F* = −1.823, *p* = 0.062, Tajima’s *D* = −0.093 NS) suggest an excess of low frequency alleles and therefore the possibility of demographic expansion cannot be rejected based on these results. Despite this, the Ramos-Onsins and Rozas for each marker were not significant (CR; *p* = 0.111, ND2; *p* = 0.087). The BSP shows a population expansion within the last 300 K. y. a. This data is in accordance with the mismatch distribution analyses and the lack of population structure as seen from the phylogenetic and network analyses.

The best-fitting models for the CR and ND2 alignments were the following; *n* = 62; HKY+G and HKY+I, *n* = 181; HKY+I+G, ND2 by codon partition, TrN+I, K80+I, HKY and *n* = 148; HKY+I+G and TrN+I. All analyses recovered a well-resolved monophyletic clade composed by Korean+Chinese individuals. The larger data sets (*n* = 181, *n* = 148) recovered three clades. The RAxML, BI and continuous Bayesian analyses recovered the Korean clade sister to the clade composed by individuals from all Chinese localities (Central, North East, South East, West China), and a more ancestral Chinese Western clade. However, the Bayesian discrete tree recovered the Korean clade as sister clade to the Western China clade, but this relationship was weakly supported. The rooted ML and BI tree for the smaller alignment (*n* = 62 + outgroup) recovered the same tree topology with two sister clades, one composed by only Korean taxa and the other by Korean and 15 Chinese individuals from four localities. The discrete MCC tree (Fig. S1) and S-DIVA analyses (Fig. 6) indicate a Korean ancestral origin of the Chinese haplotypes and therefore point to a recent expansion from the Korean Peninsula towards mainland China.

The ML networks revealed a clear lack of structure within the Korean metapopulation with most populations recovering different haplotypes with no geographical structure (Fig. 5). For the continuous analyses the Strict Gamma RRW was selected over all other tested models for the Korean and closely related Chinese individuals (reduced alignment) and the Strict Cauchy RRW for the larger alignment with all Chinese localities (*n* = 148) (Table S1). In congruence to the rooted phylogenies, the population-based discrete and continuous phylogeographic analyses recovered two clades, the Chinese-Korean and Korean. The trees showed high posterior probability support for the Chinese+Korean but poor support for the Korean clade, which recovered two strong supported clades with no apparent geographical structure. Individuals from the same localities were grouped within the different clades, potentially showing admixture of the metapopulation. The continuous MCC tree revealed a time split between these clades dating to circa 700 K. y. a. However, the limited number of samples from the mainland and the few markers available (mostly CR), resulted in modest node support, and thus, caution is needed in the interpretation of the dispersal events from the Korean Peninsula towards the mainland. With these analyses we aim to elucidate evidence on the dispersal path (Korea to China or China to Korea) or the bi-directionality of such. The projection of the MCC tree (Fig. 4) in the geographical map (Fig. 8) shows an original expansion of the Western Chinese population with an early radiation towards the Korean Peninsula at approximately 2 M. y. a. From 2 M. y. a. to about 800 K. y. a. there is an overall population expansion in Western, Central and South East China. The current population spread throughout all the Chinese localities and point to a continuous and progressive expansion of the populations. The projection of the MCC tree (Fig. S2) in the geographical map of the Korean *B. gargarizans* and closely matched Chinese individuals (Fig. 7) shows a Korean expansion circa 300 K. y. a., with a subsequent Northern expansion lineage towards South Eastern China progressing further north to North Eastern China (north of Northern Korea). The Gamma RRW model suggests this population colonised North Western China, not being a direct colonisation from the Korean Peninsula population, however this colonisation route was not recovered in the Cauchy RRW models of either the small or large continuous analyses, suggesting a direct colonisation from Korea. The South West China population was likely colonised through land bridge formations over the Yellow Sea from Central Western Korea circa 300 K. y. a. The MCC tree supports the BSP analyses in the timing of the Korean Peninsula major population expansions, dating to the around the last 200 and 150 K. y. a. (Fig. 7).

### Discussion

Although numerous amphibian species see their range restricted to the Korean Peninsula, our results emphasise a broad distribution of *B. gargarizans*, supported by the presence of common haplotypes in Korea as well as North East Asia. The Korean Peninsula served as a refugium for several species during either glacial or inter-glacial periods for anurans (Zhang et al., 2008; Yoshikawa et al., 2008), as well as for other more efficient dispersers (Hosoda et al., 2000; Kim et al., 2013; Koh et al., 2014). Our findings suggest colonisation of the Korean Peninsula by individuals originating from the Chinese mainland between 2 and 0.8 M. y. a. followed by weak isolation or repeated gene exchange, as seen through the monophyly of a Korean clade within the widespread *B. gargarizans* species complex. This was followed by dispersal events originating from the Korean Peninsula, northwards over land and westwards over land bridges during sea level falls.

The population expansion seen on the Chinese mainland, and on then Korean Peninsula, for the last 2 m.y. started during the paeloperiod when the drainage basins of the Han, Amur, Liao and Yellow Rivers were joined into a single unit, flowing into the ocean south of the current Yellow Sea (Ryu et al., 2008). At the period in the early Quaternary, the totality of the Chinese Mainland, Taiwanese and Japanese Islands and the Korean Peninsula were connected by the continental shelf for the last time (see Fig. 1, He et al., 2015). This increased landscape connectivity (Xie, Jian & Zhao, 1995; Diekmann et al., 2008) enabled toads to breed, disperse, and colonise new areas unhindered. Besides, this period matches with the establishment and reinforcement of the monsoon weather (Wang & LinHo, 2002; Lee, Jhun & Park, 2005; He et al., 2015), which also contributed to an increase of habitat suitability for amphibians, and the proliferation of populations.

During the next glacial maximum of the next 2 m.y. the Korean Peninsula may have not been isolated during glacial maxima, but during inter-glacials, as a complementary hypothesis to the four options raised by Kim et al. (2013), and explaining the unexpected ‘expansion period (0.02 ± 0.02 [m.y.a]) corresponding to the LGM’ (Kim et al., 2013). The glacial maxima resulted in a larger continuous refugium connecting the Korean Peninsula and the Chinese mainland south of the ice line (Zhan & Fu, 2011; Yan et al., 2013), due to the absence of ice in the Korean Peninsula during the LGM (Kong, 2000; Walker et al., 2009; Yi & Kim, 2010), and the drop in sea levels, accumulated in glaciers, during glacial maxima. This will have allowed for the continuous gene exchange, and the later population expansions, followed by the northward dispersion events. The potential increased connectivity between Chinese mainland and Korean Peninsula during this period are the Mindel-Riss inter-glacial (MIS 11; in Europe: Kukla, 2005; in Russia: Galuskin et al., 2009), the Riss glacial period (MIS 6; Kazantseva glaciation, Vasil’ev, 2001; Müller, Pross & Bibus, 2003) and the glaciation period called the Wurm Glaciation in Europe (=MIS 2–4; Ehlers & Gibbard, 2008; Zyryanka in Asia: Astakhov, 1998).

The recovery of two distinct clades within the peninsula reflects likely episodes of dispersal events to/from the Korean Peninsula. The median time since the most recent common ancestor (TMRC) between the two clades generally matches with the interglacial period before the Gunz Glaciation (=Marine isotope stage (MIS) 16; Schweitzer, 2004), circa 700 K. y. a., when the Korean and the mainland became isolated due to sea level resurgence. Topographical and environmental heterogeneity is more often than not a predictor to patterns of increased genetic variability sustained by different levels of allopatric diversification. Within Korea, the lack of North–South genetic structure may have been foreseen due to the continuity of low plains facilitating dispersion of anurans, as visible through the current range of *Dryophytes suweonensis* (Borzée, Yu & Jang, 2016; Borzée et al., 2017) and *P. chosenicus*. In contrast, several studies have shown the role of East-West topographical barriers in the Korean Peninsula, contributing to allopatric speciation in amphibians, such as for *Hynobius* spp. (Baek et al., 2011; Min et al., 2016) and *D. japonicus* (Dufresnes et al., 2016). However, despite the presence of landscape barriers throughout the sampled area, such as the Baekdu Mountain Range and the sea channel between the mainland and Gangwha Island, genetic exchange was not constrained, as shown by the presence of common haplotypes on both sides of these candidate geographical barriers. This pattern was unexpected due to the topographical relief of Baekdu Mountain Range above the occurrence threshold of *B. gargarizans*. We interpret this apparent lack of genetic structure as a result of efficient dispersal capabilities in *B. gargarizans*, as seen in most toads (Vences et al., 2003), or dispersion over emerged lands during glacial maxima.

*Bufo gargarizans* in Chinese localities (HLJ, HB and GS in Table 1) recovered common haplotypes for the CR in Korean individuals, implying recent dispersal and gene flow between localities. The clustering of South Korean and Chinese haplotypes in a single clade subsequently to the last major interglacial period is historically coherent due to the regular fluctuations of the Yellow Sea level following minor ice ages (Jingtai & Pinxian, 1980; Oba et al., 1991; Liu et al., 2009). The Korean Peninsula was last connected to the Chinese mainland over the Yellow Sea during the late Pleistocene (Millien-Parra & Jaeger, 1999), and species were able to take advantage of land bridges to disperse until the end of the LGM, circa 15 K. y. a. (Chung, 2007; Lomolino et al., 2010). However, we need to address that while the presence of a Korean and a Korean + mainland clade may suggest possible expansions from different areas of potentially ancestral allopatric populations that reconnected in the Peninsula, this pattern can also be caused by incomplete lineage sorting, where some individuals still retain ancestral haplotypes (i.e. partial divergence with no secondary contact). Although the idea of two disjoint populations coming into secondary contact in the peninsula sounds appealing, our data does not lend to test hypotheses of admixture, thus testing apart secondary contact from lineage sorting would be difficult (Qu et al., 2012).

The case of Gangwa Island is peculiar as the two sampled individuals were breeding males, identifiable through the unmistakable forearm muscular development, but about a third smaller than average male size, 2.83 cm (± 0.12 cm), versus 8.08 cm (± 0.67 cm) in average for males *B. gargarizans* (Cheong, Sung & Park, 2008). This size variation may be explained by the island effect, where isolated individuals drift in average size, while being genetically closely related, i.e. same haplotype, to individuals on the mainland. This is, for instance, the case of *D. japonicus* between Jeju Island and the Korean Mainland (Jang et al., 2011). Further sampling in this and other satellite islands is likely to elucidate further information on the processes of dwarfism in the area.

The topographical effects caused by sea level changes throughout the ice ages in Asia and Europe are contrasting. In Europe, ice shelves lead to the southwards displacement of species resulting in isolation through allopatric speciation on southern peninsulas (i.e. Iberian, Italian, Greek, Balkans; Hewitt, 2000). In contrast, sea level recession in North East Asia at glacial maxima resulted in the drainage of the Yellow Sea (Jingtai & Pinxian, 1980; Haq, Hardenbol & Vail, 1987; Fairbanks, 1989; Park, Khim & Zhao, 1994; Kim & Kennett, 1998; Ijiri et al., 2005; Liu et al., 2009) and the near closing of the Tsushima Straight (Oba et al., 1991; Millien-Parra & Jaeger, 1999; He et al., 2015). Land bridge formations between the mainland, the Korean Peninsula (Haq, Hardenbol & Vail, 1987; Millien-Parra & Jaeger, 1999; Chung, 2007; Lomolino et al., 2010) and the then continuous formation of the Japanese Islands effectively allowed genetic admixture between populations as seen in this study, and blurring of species boundaries through introgressive extinction.

## Supplemental Information

10.7717/peerj.4044/supp-1 Supplemental Information 1 Control region alignment raw data.

10.7717/peerj.4044/supp-2 Supplemental Information 2 ND1 alignment raw data.

10.7717/peerj.4044/supp-3 Supplemental Information 3 Concatenated alignment raw data.

10.7717/peerj.4044/supp-4 Supplemental Information 4 Supplementary Information.

## Additional Information and Declarations

### DNA Deposition

The following information was supplied regarding the deposition of DNA sequences: GenBank sequences can be found in Table 1.

### Data Availability

The following information was supplied regarding data availability: The raw data has been supplied as Supplemental Dataset Files.
