# Supplementary material for: Phylogeographic and population insights of the Asian common toad (Bufo gargarizans) in Korea and China: population isolation and expansions as response to the ice ages
Source: PeerJ. 2017 Nov 28;5:e4044. doi: 10.7717/peerj.4044 (PMC5710166; doi:10.7717/peerj.4044)
Supplement: Supplemental Information 4 [file peerj-05-4044-s004.docx]

SUPPLEMENTARY INFORMATION

**Phylogeographic and population insights of the Asian Common toad (*Bufo gargarizans)* in Korea and China: Population isolation and expansions as response to the ice ages.**

SI Figure 1. MCC discrete diffusion coalescent tree of *Bufo gargarizans*. Chinese localities are: NorthEastChina (HJ), SouthEastChina (CIB-XM), NorthWestChina (GS), SouthWestChina (HB). Nodes circles are colour coded by localities. The branch widths correspond to higher or lower posterior probabilities recovered from the analyses.


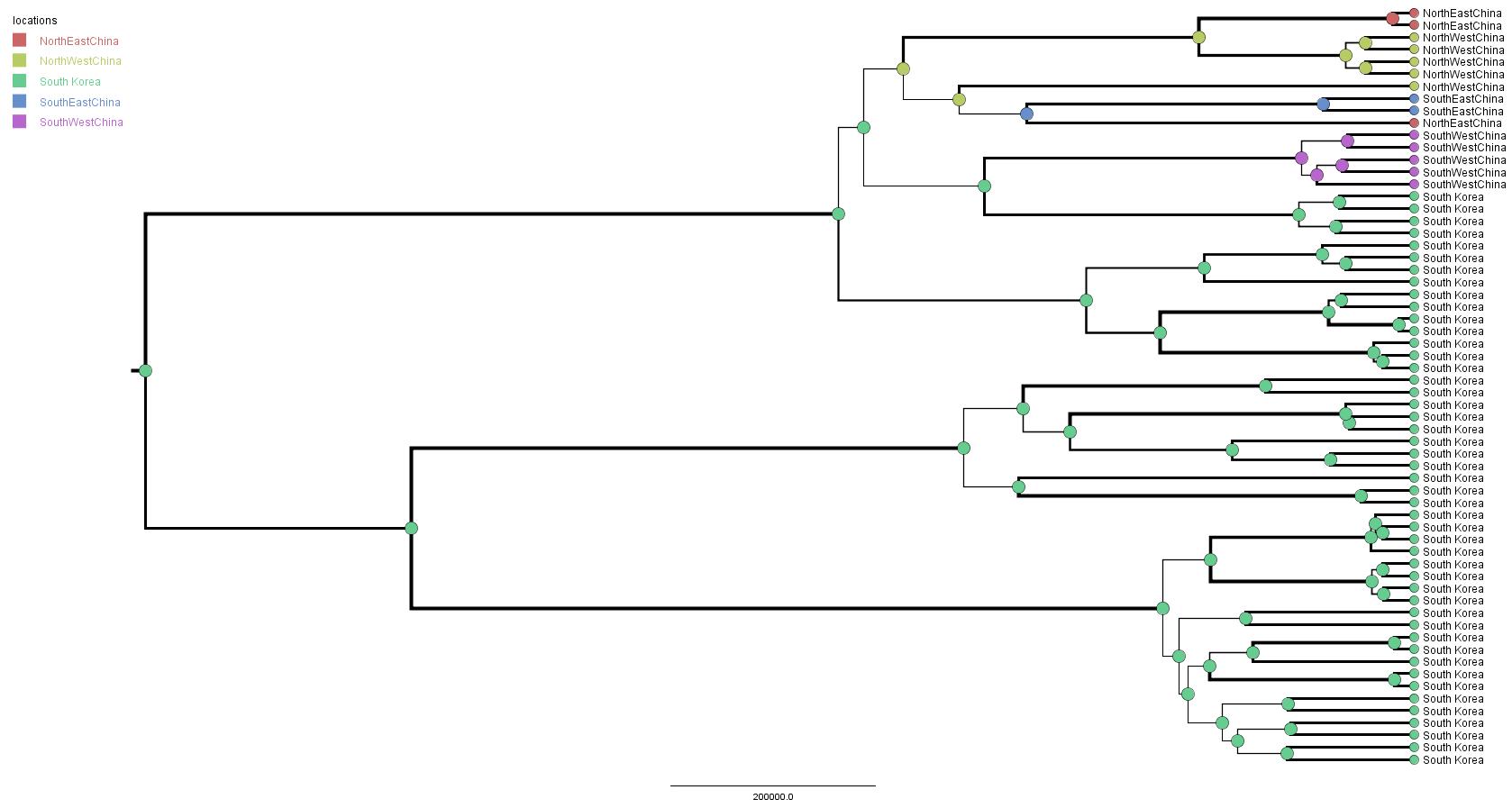


SI Figure 2. MCC continuous diffusion coalescent tree of *Bufo gargarizans*. High posterior probability nodes above >95% are represented in red circles andn branches in black.


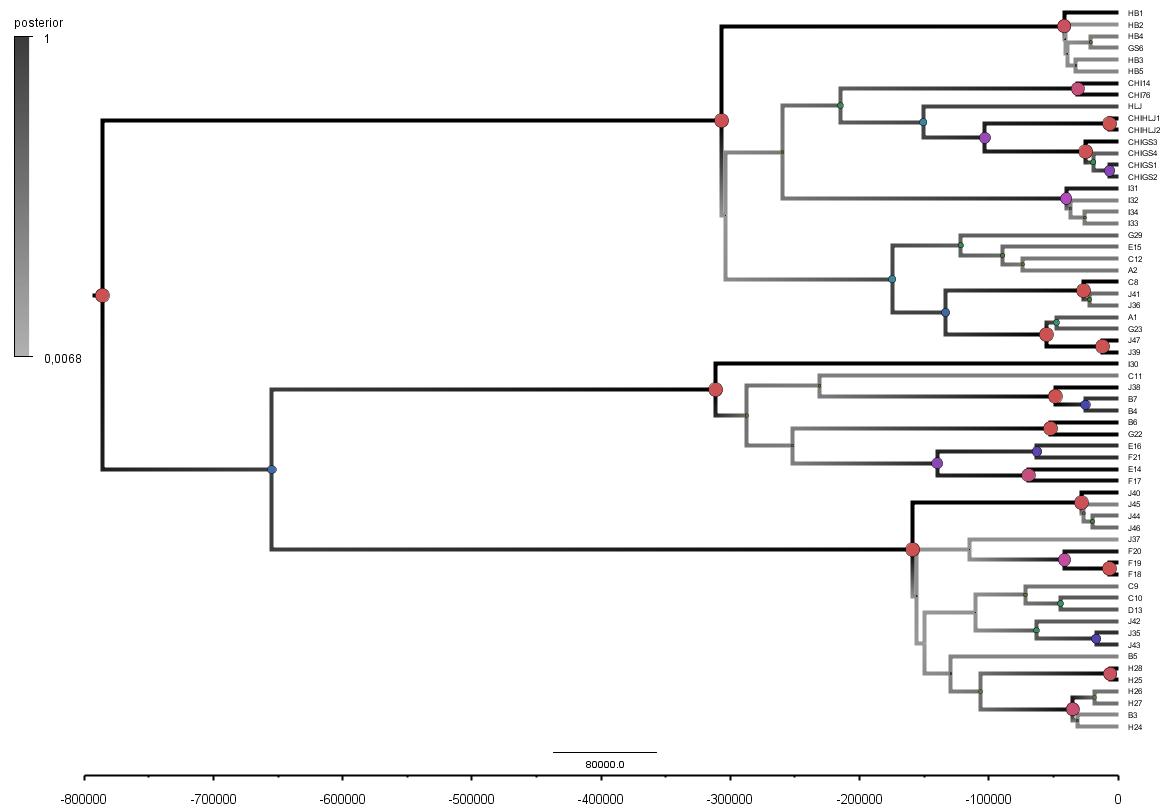


SI Figure 3. Best Maximum Likelihood tree recovered from the RAxML analyses for the ND1 and CR fragments, displaying the genetic structure of *Bufo gargarizans* from the Korean Peninsula and mainland Chinese localities. Posterior probabilities > 0.95 from the Bayesian Inference are indicated on nodes. The outgroup is *B. tibetanus* (accession number JX878885).


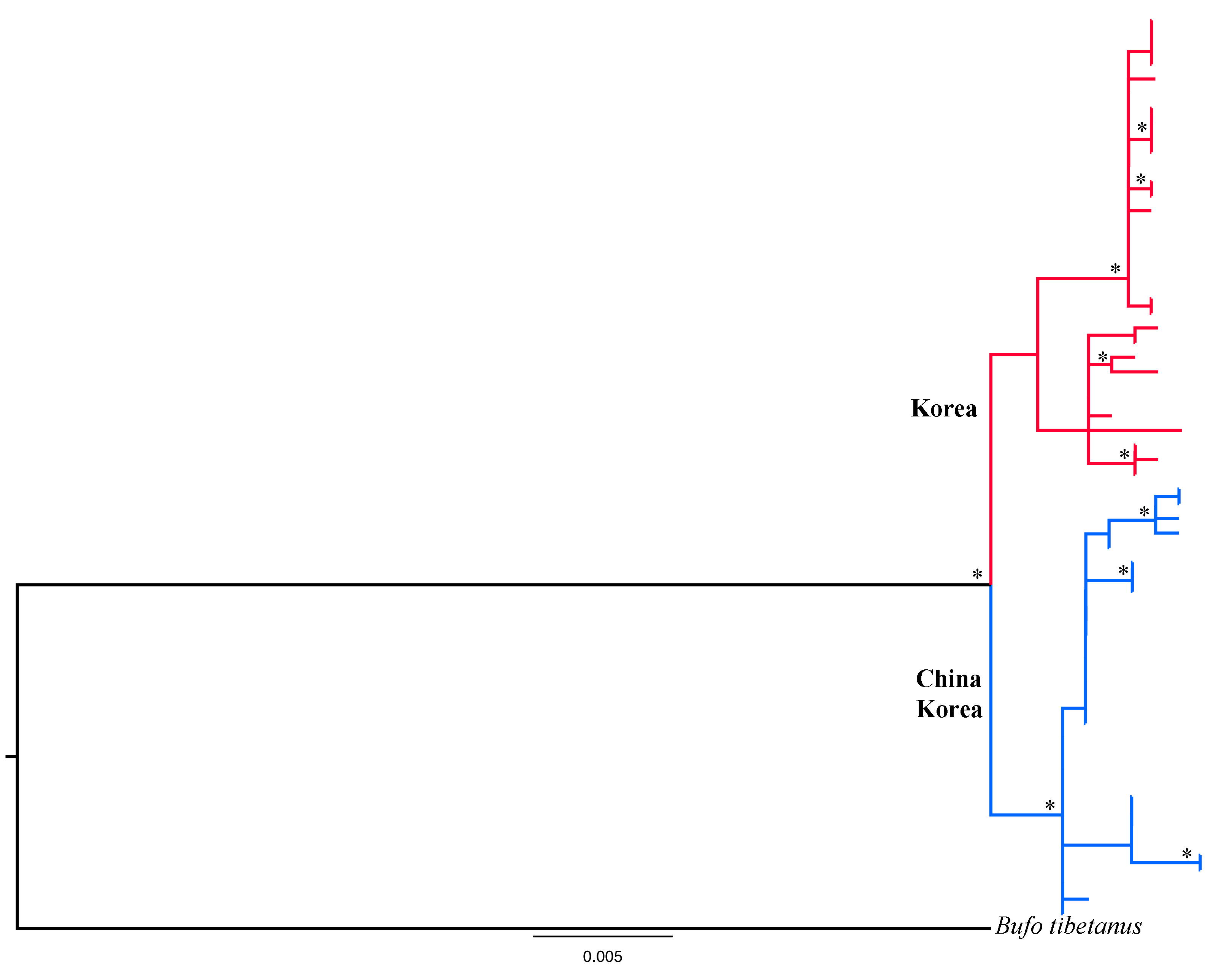


SI Figure 4. The unimodal mismatch distribution of the Korean (n=47) and Chinese closely related *Bufo gargarizans* (n=15). (A) ND1 mismatch distribution, (B) CR mismatch distribution.


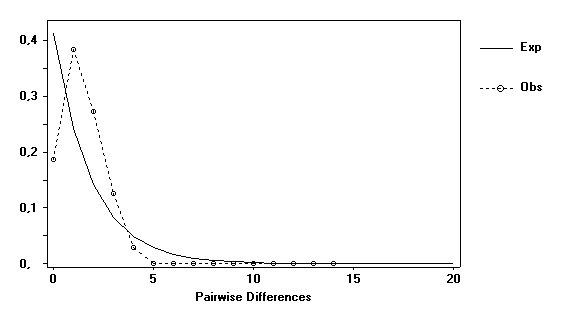

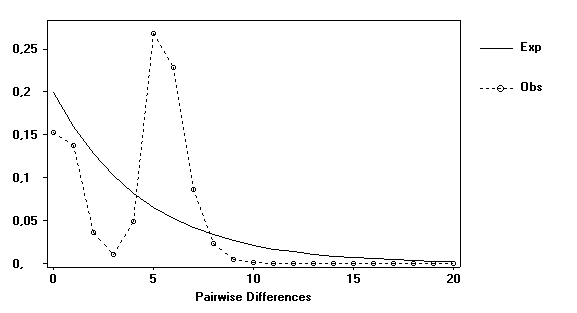


(A)

(B)

## SM Table 1. Marginal likelihood estimates and Bayes factor comparison of coalescent priors for *Bufo gargarizans*. The asterisk (*) represents the best model selected for each alignment.


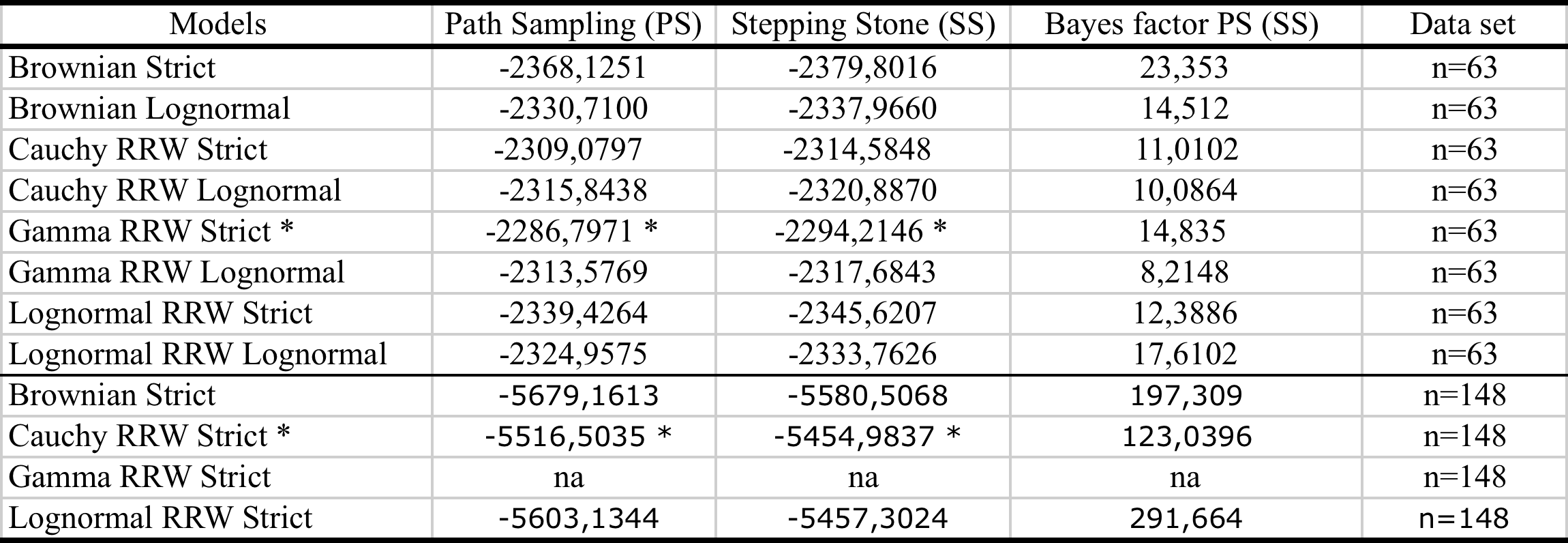


na: Gamma RRW non convergence of ESS >50.
